# A Comparative Study of Retrorectus Mesh Placement Versus Properitoneal Mesh Placement in Open Repairs of Ventral Hernias

**DOI:** 10.7759/cureus.45277

**Published:** 2023-09-15

**Authors:** G Akhil Chowdari, Narayanaswamy Chetty Y V, Nandakumar BM

**Affiliations:** 1 General Surgery, Ramaiah Medical College, Bengaluru, IND; 2 General Surgery, Indira Gandhi Medical College, Shimla, IND

**Keywords:** hernioplasty, properitoneal mesh repair, retrorectus mesh repair, mesh repair, ventral hernia

## Abstract

Background

Ventral hernias affect millions of patients each year. Surgery is the main line of management and various techniques have been advocated; however, mesh repair has become the norm and different approaches have been described regarding the plane of mesh fixation, but none of them are standardized. Open repair is commonly practiced, and the two most commonly performed methods are retrorectus and properitoneal mesh placement.

Objectives

To compare the postoperative outcomes between the retrorectus plane and the properitoneal plane of fixation of mesh in open ventral hernia repair.

Methods

Between September 2018 and August 2020, 56 patients with midline ventral hernia admitted to Ramaiah Hospital, Bengaluru were chosen for this prospective comparative study. Group A had 28 patients who underwent open retrorectus mesh repair and 28 patients in Group B underwent open properitoneal mesh repair. The postoperative outcomes were studied in terms of operating time, postoperative complications, and early recurrence at the end of six months and 24 months post-surgery.

Results

The operative time for retrorectus mesh placement was significantly lower than properitoneal mesh placement. The latter had a higher complication rate overall with an incidence of 18%, with seroma being the most common complication; however, the difference in complication rates was not statistically significant. Skin necrosis was identical in both groups and 0% of cases in both groups had SSI or mesh infection. Three patients (10.71%) in the retrorectus group and two patients (7.10%) in the properitoneal group developed recurrence at 24 months follow-up.

Conclusion

Retrorectus mesh repair and properitoneal mesh repair in open ventral abdominal hernias have equally good postoperative outcomes.

## Introduction

Hernias of the anterior abdominal wall, or ventral hernias, may be congenital or acquired, and represent defects in the parietal abdominal wall fascia and muscle through which intra-abdominal or preperitoneal contents can protrude [1]. The most common ventral hernias are the incisional and paraumbilical hernias which constitute about 85% of overall ventral hernias [2]. Surgery is the main line of management. There is a high chance of recurrence following ventral hernia surgeries when compared to inguinal hernia surgery. Over the years, surgeons all over the world have tried various techniques for the repair of ventral hernias; while each procedure had its own advantages and disadvantages, meshplasty was considered to be the best. Subsequently, it was realized that the placement and fixation of the mesh were crucial in determining the outcome of the repair [3]. 

Laparoscopic hernia repair has become the standard of care for ventral hernia. It has the advantage of less pain, small wounds, lower chances of surgical site infection (SSI), shorter hospital stays, and early recovery. Laparoscopic hernia repair has a steep learning curve with the higher cost of equipment as well as mesh and fixation devices being the major disadvantages. In a resource-restricted setting, open hernia repair is preferred over laparoscopic hernia surgery.

Several mesh repair techniques have been developed including the placement of onlay mesh (over the fascia), inlay mesh (a bridging repair of the fascial defect with little or no fascial overlap), retrorectus mesh (sublay), properitoneal mesh, and underlay mesh. Recurrence rates after open repair of up to 20% have been reported and are predisposed by mesh size and fixation type [4].

Properitoneal meshplasty is one of the most common open procedures that has been advocated for ventral hernias but numerous studies support the retrorectus approach due to its easier approach and better outcomes. Hence, a study to compare retrorectus meshplasty and properitoneal meshplasty was done.

## Materials and methods

Objective of the study

To study and compare the postoperative outcome between retrorectus mesh placement and properitoneal mesh placement in ventral midline hernias.

Study design

This prospective comparative study included all patients diagnosed with midline ventral hernia undergoing open mesh repair who met the inclusion and exclusion criteria from the start of the study until the sample size was achieved.

This study was conducted in Ramaiah Medical College and Hospitals after receiving ethical approval from the Ethics Review Board, Ramaiah Medical College and Hospitals (ERB registration number - ECR/215/Inst/KA/2013/RR-22) with approval number EC/PG-33/2018.

To achieve results that can be statistically analyzed, considering an effect size of 0.8 and mean difference of 0.4 between the groups, considering the power of 80% and alpha error of 5%, the sample size was calculated to be a minimum of 28 in each group, with a total sample size of 56.

Inclusion and exclusion criteria

Patients of both sexes aged 18 years and above with diagnosed midline ventral hernia who planned to undergo open mesh hernioplasty were included in the study and followed up as per protocol for 24 months.

Patients presenting with complications of ventral hernia (intestinal obstruction, incarceration, strangulation), pregnant patients, patients with recurrent hernias, and patients who were not willing to participate in the study were excluded.

Methodology

A total of 56 patients diagnosed with midline ventral hernia were included in the study. The included patients were subjected to complete history taking of preoperative symptoms and the mode of presentation was recorded after clinical examination. After confirmation of diagnosis, all the patients' hemograms, coagulation profile liver function tests, and renal function tests were conducted, after which they underwent pre-anesthetic checkups. From these, 28 patients included in Group A underwent retrorectus meshplasty where the polypropylene mesh fixed the posterior to rectus muscle and anterior to posterior rectus sheath with Prolene 2-0 sutures, and 28 patients in Group B included properitoneal meshplasty where the polypropylene mesh fixed posterior to posterior rectus sheath and anterior to peritoneum with Prolene 2-0 sutures; the size of the defect, polypropylene mesh size, and operating time were recorded respectively.

In all cases vertical midline incision was made. The sac of the hernia was identified and dissected free from the neck, subcutaneous tissue contents were reduced and the sac was either excised or reduced. Vicryl No1 suture material was used for the closure of the peritoneum and posterior rectus sheath with a continuous running suture. An appropriate size mesh was placed in the properitoneal or retrorectus plane. Polypropylene mesh was fitted in order to exceed the edge of the defect at least 5cm in all directions. The mesh was fixed to Prolene no 2-0 with transfascial fixation. The anterior rectus sheath was closed with a running suture using Prolene no 1. A negative suction drain was placed in the subcutaneous plane if wide subcutaneous dissection was done and not in all cases. The postoperative outcome of all patients was assessed using the following parameters: postoperative pain according to a visual analog scale (VAS) which ranges from 1-10, presence of drain, duration of drain kept, postoperative complications like seroma, skin necrosis, SSI, and mesh infection.

The patients were followed up for short-term post-discharge from the hospital for suture removal between postoperative Days 8 to 14 and the status of the wound and complications (if present) were recorded. Long-term follow-ups were conducted at three-, six-, and 24-month intervals, and any recurrence was recorded. If any suspicion was found, then the patient was requested to undergo soft tissue scans for confirmation of recurrence.

Statistical analysis

Data were entered into a Microsoft Excel spreadsheet and analyzed using SPSS v.22 software (IBM Corp., Armonk, NY). Categorical data were presented in terms of frequencies and proportions. The chi-square test or Fischer's exact test (only for 2x2 tables) was used as the significance test for qualitative data. Continuous data were presented as mean and standard deviation. The independent t-test was used as a significance test to determine the mean difference between two quantitative variables. MS Excel and MS Word were used to obtain different types of charts. A p-value of <0.05 was considered statistically significant after accepting all rules of statistical tests. SPSS software was used for data analysis.

## Results

Results

A total of 56 patients were included in the study. Each group had 28 patients and the demographic data in both the groups were comparable. Most of the cases were in the age group 31-40 years and 51-60 years with an increase in the incidence of ventral hernias from the third to sixth decade of life.

The male to female ratio was 0.9 with a total of 29 (51.78%) males and 27 (48.21%) females. Among males, the age group of 51-60 years had the highest presentation while in females the most common age group was between 31-40 years.

The most common comorbidity was hypertension 14 (25%) when compared to other comorbidities like diabetes, ischemic heart disease, asthma, and other comorbidities like hypothyroidism, COPD, and chronic kidney disease; 32 (57.14%) patients didn’t have any comorbidities (Table 1).

**Table 1:** Patient demographics N: number of patients; SD: standard deviation; IHD: ischemic heart disease p-value of <0.05 is considered significant

Parameter		Retrorectus		Properitoneal		P-value
		Number	Percentage and SD	Number	Percentage and SD	
Mean age (years)		48.43	SD±13.20 years	49.11	SD±13.806 years	0.852
Sex	Male	15	53.60%	14	50.00%	0.789
	Female	13	46.40%	14	50.00%	0.789
Mean BMI (kg/m^2)^		27.047	SD±4.182	27.241	SD±5.64	0.884
Comorbidities	Hypertension	7	25.00%	7	25.00%	1
	Diabetes	6	21.40%	3	10.70%	0.469
	IHD	1	3.60%	3	10.70%	0.611
	Asthma	2	7.10%	1	3.60%	0.553
	Others	0	0.00%	3	10.70%	0. 236
Type of hernia	Epigastric	2	7.10%	4	14.30%	0.025
	Paraumbilical	22	78.60%	11	39.30%	
	Incisional	4	14.30%	12	42.90%	
	Spigelian	0	0.00%	1	3.60%	

The mean BMI among subjects with retrorectus meshplasty was 27.047±4.182 kg/m2 and the mean BMI among subjects with properitoneal meshplasty was 27.241±5.640 kg/m2 (Table 1). There was no statistically significant difference found between the two groups with respect to BMI.

Paraumbilical hernias were the most common type of ventral hernia with a distribution of 59% (N=33) of cases and incisional hernia was the second-most common with 29% (N=16) of cases.

The most common hernia noted were paraumbilical hernias among both males and females (Table 1). Incisional hernia was more common among females with a female-to-male sex ratio of 3:1. Epigastric hernia was seen only in males while spigelian hernia was noted only in females. The paraumbilical hernia had preponderance for the male sex with a ratio of 1.35:1. There was a statistically significant difference found between the two groups with respect to the type of hernia.

The mean duration of surgery for the retrorectus mesh hernioplasty group was 95.89±23.335 minutes and for the properitoneal mesh hernioplasty group was 110.89±29.252 minutes (Table 2). There was a statistically significant difference found between the two groups with respect to the duration of surgery. The majority of 39 (69.64%) cases had a defect size between 2-4 cm with a comparable number of cases undergoing retrorectus and properitoneal repair with the maximum size of a defect being 6 cm (Table 3). As depicted in Tables 4-5, as the size of the hernia defect increased there was a proportional increase in the duration of surgery for properitoneal hernia repair.

**Table 2:** Operative results and complications N: number of patients; SD: standard deviation; POD: postoperative day; SSI: surgical site infection p-value of <0.05 is considered significant

	Parameter	Retrorectus		Properitoneal		P-value
		Number	Percentage and SD	Number	Percentage and SD	
Mesh size used	7.5x15cm	20	71.40%	15	53.60%	0.269
	15x15cm	8	28.60%	13	46.40%	
	Mean defect size	2.714	SD+/-1.2051	3.018	SD+/-1.3641	0.381
	Duration of surgery	95.89	23.335	110.89	29.252	0.039
Pain VAS	POD1	4.61	0.79	4.57	1.07	0.887
	POD2	3.18	1.02	3.29	1.08	0.705
	POD3	2	0.9	2.18	0.98	0.482
	POD4	1.54	0.58	1.54	0.69	1
	POD5	1.18	0.39	1.29	0.53	0.395
	Duration of drain use	2.75	2.03	3.18	2.722	0.507
	Duration of hospital stay	5.18	3.232	4.89	2.149	0.699
	Seroma	2	7.10%	4	14.30%	0.669
	Skin necrosis	1	3.60%	1	3.60%	1
	SSI	0	0%	0	0%	
	Mesh infection	0	0%	0	0%	
Recurrence	3 months	0	0%	0	0%	
	6 months	2	7.10%	1	3.57%	0.8504
	24 months	3	10.71%	2	7.10%	

**Table 3:** Distribution of cases according to defect size and mesh plane fixation N: Number

HERNIA DEFECT CM	RETRORECTUS REPAIR	PERCENTAGE	PROPERITONEAL REPAIR	PERCENTAGE	TOTAL
0-2	2	7.14%	2	7.14%	4
2-4	20	71.42%	19	67.85%	39
>4 cm	6	21.42%	7	27%	13
TOTAL	28		28		56

**Table 4:** Duration of surgery and type of repair N: Number

TIME (min)	RETRORECTUS	PERCENTAGE	PROPERITONEAL	PERCENTAGE
60-89	8	28.57%	3	10.71%
90-119	13	46.42%	13	46.42%
120-149	6	21.42%	8	28.57%
150-180	1	3.57%	4	14.28%
TOTAL	28		28	

**Table 5:** Duration of surgery according to type of repair and defect size N: Number

	60-90 min			90-120 min			120-150 min			150-180 min			TOTAL
	0-2 cm	2-4 cm	> 4 Cm	0-2 cm	2-4 cm	> 4 Cm	0-2 cm	2-4 cm	> 4 Cm	0-2 cm	2-4 cm	> 4 Cm	
RETRORECTUS	1	5	2	1	11	1	0	4	2	0	0	1	28
PERCENTAGE	3.57%	17.85%	7.14%	3.57%	39.28%	3.57%	0%	14.28%	7.14%	0%	0%	3.57%	
PROPERITONEAL	1	2	0	1	12	0	0	4	4	0	1	3	28
PERCENTAGE	3.57%	7.14%	0%	3.57%	42.85%	0%	0%	14.28%	14.28%	0%	3.57%	10.71%	

**Figure 1:**
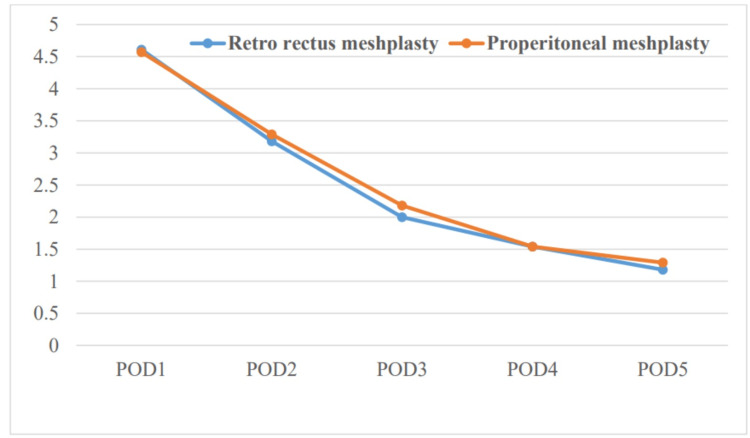
Graph showing the comparison of the pain VAS score between the two groups VAS: visual analogue scale; POD: postoperative day

The pain scores are comparable in both groups with no significant pain at the end of Day 4 requiring injectable analgesics as shown in Figure 1. There was no statistically significant difference found between the two groups with respect to pain VAS on postoperative Days 1-5.

Around 7% (N=2) cases of retrorectus and 14% (N=4) of properitoneal repair had seromas that were treated conservatively with aspiration. Skin necrosis was seen in both groups equally at 4% (N=1) incidence (Table 2).

Drain insertion was decided based on the amount of subcutaneous dissection and the size of the defect. Around 75% (N=21) of cases in the retrorectus group and 68% (N=19) of cases in the properitoneal group had a drain in situ, which in the majority of cases was removed between the third and fourth postoperative day for the retrorectus group and between the fifth and sixth day for the properitoneal group when the drain output was less than 20 ml. One (3.57%) case in the retrorectus group and two (7.14%) cases in the properitoneal group had prolonged drain in situ due to extensive dissection and obesity of the patient.

Around 80% (N=45) of cases were discharged in less than five days after surgery of which 53% (N=24) cases were in the retrorectus group and 47% (N=21) were in the properitoneal group (Table 2). Two cases in the properitoneal group had seromas because of which patients were discharged between the ninth and tenth postoperative day and one case in the retrorectus group was discharged on the 20th postoperative day due to intestinal obstruction which resolved with conservative management.

No recurrence was noted at the end of three months in both groups. Two patients(7.10%) in the retrorectus group and one patient (3.57%) in the properitoneal group developed recurrence at six months. Three patients (10.71%) in the retrorectus group and two patients (7.10%) in the properitoneal group developed recurrence at 24 months follow-up (Table 2).

## Discussion

Ventral hernias are one of the common cases faced by a general surgeon in their regular practice. Ventral hernias belong to a broad group of hernias occurring in the anterior abdominal wall which include the following: epigastric, paraumbilical, spigelian, and incisional hernias. The standard approach towards the management of ventral hernia is that of fascial defect repair and mesh reinforcement of the abdominal wall. In the last two decades, ventral hernia repair has undergone significant changes with respect to approach, choice of mesh, and selection of mesh plane. The focus of this study was to compare the outcomes with regards to the selection of mesh planes with reference to retromuscular placement or retrorectus plane and retrofascial placement or properitoneal plane.

The aim of a mesh hernia repair surgery is to provide a strong, mobile, and physiologically dynamic barrier wall to prevent fascial weakness and recurrence.

The Rives-Stoppa (RS) hernia repair is a worldwide accepted open technique wherein the prosthetic mesh is placed in a preperitoneal-sublay fashion with >5cm of overlapping of the mesh beyond the hernia defect. It has the advantage of being a tension-free repair as well as providing maximum surface area for tissue ingrowth through the mesh. The original description of Stoppa repair was followed for inguinal hernias with prosthetic mesh placement in the intraparietal plane below the arcuate line, superficial to the peritoneum and deep to the transversalis fascia. A later modification of this technique for ventral hernias fixes the prosthesis posterior to the rectus abdominis muscles and anterior to the posterior rectus sheath known as retrorectus mesh repair. These techniques, popularized more recently by Stoppa and colleagues, achieve three major goals of herniorrhaphy: (1) extensive overlap between the prosthesis and the fascial edges allows a tension-free closure as well as a large surface area for tissue incorporation; (2) the mechanical strength of the synthetic prosthesis reinforces the abdominal wall, especially when there is increased intraabdominal pressure; and (3) placement of the prosthesis adjacent to the vascular-rich rectus muscles facilitates tissue incorporation, promotes resistance to mesh infection, and allows interposition of autologous tissue between the prosthesis and the skin/subcutaneous tissues anteriorly and the peritoneum posteriorly [5].

Preperitoneal mesh repair was described in literature also as a modification of the RS technique but the mesh here is placed between the peritoneum and the posterior rectus fascia, instead of between the rectus muscle and the posterior rectus fascia. Developing the preperitoneal plane is often challenging when the abdominal wall layers have been entered previously in a prior laparotomy, but it can usually be dissected sufficiently to fully protect the underlying bowel from the mesh. It has the advantage of allowing a wide mesh overlap of the defect of 8 to 10 cm or more and dissection deep into the pelvis and space of Retzius to the pubis, and the preperitoneal plane usually is devoid of any major vessels and allows for a bloodless dissection. The retrorectus mesh repair is limited at the linea semilunaris where the fascias of lateral abdominal muscles condense to re-form the anterior and posterior rectus sheath. The preperitoneal plane dissection allows the surgeon to position and safely secure the mesh to the pubis and Cooper’s ligaments, overlying the wings of the iliac bones, posterior-laterally to the psoas muscles, and beyond the costal margins superiorly. Compared to the retrorectus plane, the properitoneal plane is much thinner and more fragile leading to multiple tears if not handled with due care.

The overall sex ratio distribution of ventral hernias showed that 52% (N=29) were males and 48% (N=27) were females with a sex ratio of 0.9 showing male preponderance. Male dominance was reported by similar studies conducted by Sultan et al. [6] and Malik et al. [7].

A study by Salameh et al. [8] showed that epigastric hernias are more frequent in men with a male-to-female ratio of 3:1 and the commonly involved age group is in the third to fifth decade of life with a decreasing trend after the sixth decade, which corresponds to our study.

In our study, the age of presentation, sex distribution, BMI, and comorbidities among the retrorectus meshplasty and properitoneal meshplasty groups did not have any statistical significance which could affect the outcome of the surgery. The average defect size in the retrorectus group was 2.714 ± 1.205 cm and the properitoneal group was 3.018 ± 1.364 cm.

The time taken to execute retrorectus mesh repair was 95.89 ± 23.33 minutes while properitoneal mesh repair was 110.89 ± 29.25 minutes. There was a significant statistical difference between the groups with a p-value of 0.039. This could be attributed that the retrorectus plane is a virgin plane and easier to manage as it’s a bloodless dissection while the properitoneal is a challenging plane to manage due to the possibility of tears in the peritoneum in a violated abdomen i.e. incisional hernias due to adhesions. The average defect size in the retrorectus group was 2.714 ± 1.205 cm and in the properitoneal group was 3.018 ± 1.364 cm with no statistical significance signifies that defect size doesn’t affect the plane of mesh fixation.

The postoperative outcomes were studied in both groups and there were no significant differences in pain scores between the two groups with mean pain scores on postoperative Day 3, being 2.0 ± 0.9 in the retrorectus group and 2.18 ± 0.98 in the properitoneal group. This can be explained that both are similar kinds of surgeries and involved similar dissections of fascia with differences in mesh placement which did not affect the pain outcomes during immediate and late post-operative periods. The presence of drains was subject to the amount of subcutaneous fat and dissection in the subcutaneous plane which did not affect the surgical postoperative outcomes of patients in both groups.

The overall complications rate in retrorectus group was 11% (N=3) with seroma being 7% (N=2) and skin necrosis being 3.6% (N=1) and properitoneal group was 18% (N=5) with seroma being 14% (N=4) and skin necrosis being 3.6% (N=1). A systematic review conducted by Frank et al. [9] which involved 743 cases of retrorectus mesh placement reported an overall complication rate of 17% and seroma rates of 3% which was comparable to our study. A decade-long prospective observational study on properitoneal ventral hernia repair conducted by Heniford et al. [10] reported an overall wound complication to be 27.3% with an average duration of postoperative stay being five days while Novitsky et al. [11] reported to be at 12.5%. The mean duration of postoperative stay in the retrorectus group was 5 ± 3 days while the duration in the properitoneal group was 5 ± 2 days, however, this observation was not statistically significant. In our study, SSI, and mesh infections were not observed in any group suggesting that both techniques are equally effective as mesh in both the techniques is below the muscular component and it prevents in transmission of infection from subcutaneous tissues to mesh.

Early recurrence within three months was not noted in either group. Two patients (7.10%) in the retrorectus group and one patient (3.57%) in the properitoneal group developed recurrence at six months. Three patients (10.71%) in the retrorectus group and two patients (7.10%) in the properitoneal group developed recurrence at 24 months follow-up. Although there was a higher recurrence rate in the retrorectus group than in the properitoneal group, the difference was not statistically significant.

Limitations of the study

In the era of laparoscopic and minimally invasive surgery, the indications of open hernia repair are minimal. One of the main constraints to laparoscopic hernia surgery, apart from the long learning curve, is the cost of mesh and fixation devices to the patients. In a resource-poor country, patients opt more for a cost-effective surgery than a cosmetically better one.

Our study concludes with the results obtained over a two-year follow-up. A longer follow-up period is required for continued analysis.

## Conclusions

In conclusion, there is no accepted consensus in open hernia surgery on the ideal technique for the management of ventral hernias. Sublay is accepted to be the best plane for mesh placement but there is minimal data on whether retrorectus or properitoneal mesh repair is ideal. Our study provides a comparative analysis of two techniques commonly used in open ventral hernia repair. Our findings show that apart from the duration of surgery which was shorter in the retrorectus than in properitoneal repair, there was no significant difference in terms of duration of hospital stay, postoperative complications, or recurrence in patients undergoing retrorectus mesh repair and properitoneal mesh repair for open ventral hernia. Retrorectus and properitoneal mesh repair are safe and effective with similar outcomes. A larger study sample and longer follow-up periods may be required to establish a difference in outcomes in further studies.
